# Combined use of ursodeoxycholic acid and bosentan prevents liver toxicity caused by endothelin receptor antagonist bosentan monotherapy: two case reports

**DOI:** 10.1186/1752-1947-8-250

**Published:** 2014-07-11

**Authors:** Tomoki Ito, Yoshio Ozaki, Yonsu Son, Tohru Nishizawa, Hideki Amuro, Akihiro Tanaka, Takeshi Tamaki, Shosaku Nomura

**Affiliations:** 1 First Department of Internal Medicine, Kansai Medical University, 2-5-1 Shin-machi, Hirakata City, Osaka 573-1010, Japan

**Keywords:** Bosentan, Liver toxicity, Pulmonary arterial hypertension, Ursodeoxycholic acid

## Abstract

**Introduction:**

Pulmonary arterial hypertension is a fatal disease characterized by progressive remodeling of the pulmonary arteries and an increase in pulmonary vascular resistance. Up to 50% of patients with systemic sclerosis have pulmonary arterial hypertension, which significantly affects the prognosis. The endothelin receptor antagonist bosentan is used for the treatment of pulmonary arterial hypertension and shows a great beneficial effect. However, the most frequent side effect of bosentan is liver toxicity, which often requires dose reduction and discontinuation.

**Case presentation:**

We report two cases (a 64-year-old Japanese woman and a 69-year old Japanese woman) of systemic sclerosis, both with severe Raynaud’s phenomenon and pulmonary arterial hypertension. Both patients had initially received bosentan monotherapy, which caused liver toxicity as indicated by increased levels of alanine aminotransferase, alkaline phosphatase, and gamma-glutamyltransferase. After dose reduction or discontinuation of bosentan, these liver function abnormalities were normalized and the patients subsequently received retreatment with a combination of bosentan and ursodeoxycholic acid. The results of liver function tests did not show any abnormalities after this combination therapy.

**Conclusions:**

These reports suggest the usefulness of ursodeoxycholic acid for preventing liver toxicity caused by bosentan. Thus, the addition of ursodeoxycholic acid to the treatment protocol is expected to be useful when liver toxicity emerges as a side effect of bosentan.

## Introduction

Pulmonary arterial hypertension is a debilitating disease characterized by progressive remodeling of the pulmonary arteries with proliferation of fibrous tissue in the vessel walls, leading to death caused by right ventricular failure. Approximately 15 to 50% of patients with systemic sclerosis have pulmonary arterial hypertension, which significantly affects the prognosis [1,2]. There is increasing evidence that endothelin-1 plays a pathogenic role in pulmonary arterial hypertension [3]. Several randomized clinical trials have demonstrated the great efficacy of endothelin receptor antagonists in pulmonary arterial hypertension; thus bosentan, as the first endothelin receptor antagonist to be marketed, was approved for the treatment of pulmonary arterial hypertension in the USA and Canada in 2001 by the Food and Drug Administration and in Japan in 2005. Bosentan has been reported to be effective for pulmonary arterial hypertension, establishing its position as a novel treatment [4,5], and has recently been approved for the prevention of digital ulcers in systemic sclerosis in Europe.

The most frequent severe side effect of bosentan is known to be liver toxicity [4-8]. The package insert of bosentan indicates that the incidence of liver toxicity is 14.3% in Japanese clinical studies and 11% in US clinical studies [9]. The incidence reported from post-marketing surveillance in European clinical studies shows annual increased transaminase levels of 10.1% in 4623 cases, with interruption of bosentan administration being required in 3.2% of cases [7]. In addition, a Phase III placebo-controlled study for 16 weeks showed 9% liver toxicity, which is dose-dependent [5]. The therapeutic options for pulmonary arterial hypertension would be expanded if we could find a means to limit liver toxicity.

In the presence of liver toxicity, a dose reduction or discontinuation of bosentan needs to be considered. Although ursodeoxycholic acid can be efficacious against liver injury in clinical practice, there are no published reports of its usefulness in combating bosentan-induced liver toxicity. The incidence of liver toxicity appears to be decreased when ursodeoxycholic acid is simultaneously started with bosentan, but definite evidence of this efficacy has not previously been established.

Two of our patients showed liver toxicity after initial bosentan monotherapy but not with the subsequent addition of ursodeoxycholic acid to their treatment protocol. This is the first report that suggests the usefulness of ursodeoxycholic acid combination therapy to prevent liver toxicity caused by bosentan.

## Case presentations

### Case 1

**Figure 1:**
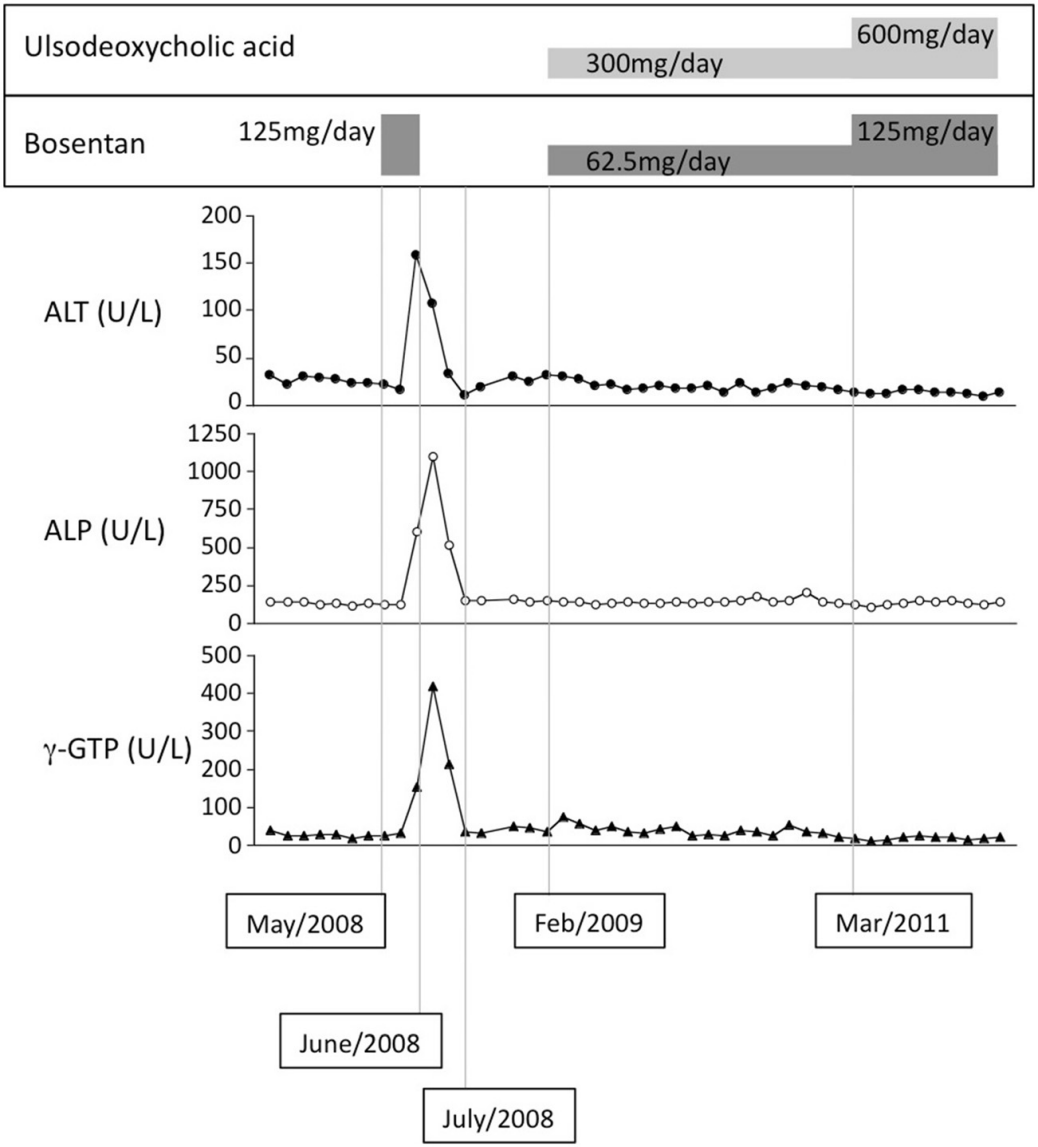
**Clinical course of Case 1.** Bosentan monotherapy was started in May 2008 at 125mg/day, and after a month, liver function abnormalities appeared. Abnormal results of liver function tests were normalized within a month after the discontinuation of bosentan. In February 2009, combination therapy of bosentan (62.5mg/day) and ursodeoxycholic acid (300mg/day) was started. There were no subsequent abnormal results of liver function tests, and the dose of both ursodeoxycholic acid and bosentan were increased to 600mg/day and 125mg/day from March 2011, respectively. No abnormal results of liver function tests were observed. Abbreviations: ALP, alkaline phosphatase; ALT, alanine aminotransferase; GGT, gamma-glutamyltransferase.

A 64-year old Japanese woman who presented with edema around her fingers and forearm followed by rapid development of skin sclerosis was diagnosed with systemic sclerosis. She was anti-topoisomerase I (Scl-70) antibody positive and had 35mmHg of estimated right ventricular pressure, as shown by echocardiography in 2006. She was prescribed prednisolone at 7.5mg/day. She was negative for both hepatitis B surface antigen (HBs Ag) and hepatitis C virus antibody (HCV Ab). In 2007, dyspnea at exertion and severe Raynaud’s phenomenon were confirmed. The findings of a chest computed tomography scan showed bilateral pulmonary fibrotic lesion and an elevated KL-6 level of 4360U/mL; she was diagnosed with pulmonary fibrosis. In addition, echocardiography results showed an elevation of her estimated right ventricular pressure to 50mmHg; she was therefore clinically diagnosed with pulmonary arterial hypertension (World Health Organization, WHO, functional class III) associated with connective tissue disease-derived pulmonary fibrosis.After bosentan treatment was initiated in May 2008 at 125mg/day, dyspnea at exertion improved and her 6-minute walking distance was increased by 40m from 332m; however, the results of blood tests indicated liver function abnormalities: increased levels of alanine aminotransferase (ALT; 4.5 times the normal upper limit), alkaline phosphatase (ALP; 3.2 times the normal upper limit), and gamma-glutamyltransferase (GGT; 6.4 times the normal upper limit; Figure 1). No other additional drugs were administered during the 2 months before and after the bosentan administration. An ultrasound examination of her liver revealed no morphologic abnormality. The abnormal levels revealed in the liver function tests were normalized soon after the discontinuation of bosentan; however, dyspnea at exertion gradually deteriorated, and therefore bosentan 62.5mg/day and ursodeoxycholic acid 300mg/day were started simultaneously from February 2009. The results of her liver function tests did not show any abnormalities even after 2 years of the combination therapy, and the dose of both ursodeoxycholic acid and bosentan were increased to 600mg/day and 125mg/day, respectively from March 2011. No abnormal levels were found in the results of her liver function tests during 31 months of follow-up after the dose elevation of bosentan. Bosentan improved her estimated right ventricular pressure to 35mmHg, subjective symptoms from WHO functional class III to II, and Raynaud’s phenomenon after bosentan retreatment, and she is currently receiving bosentan as an out-patient.

### Case 2

**Figure 2:**
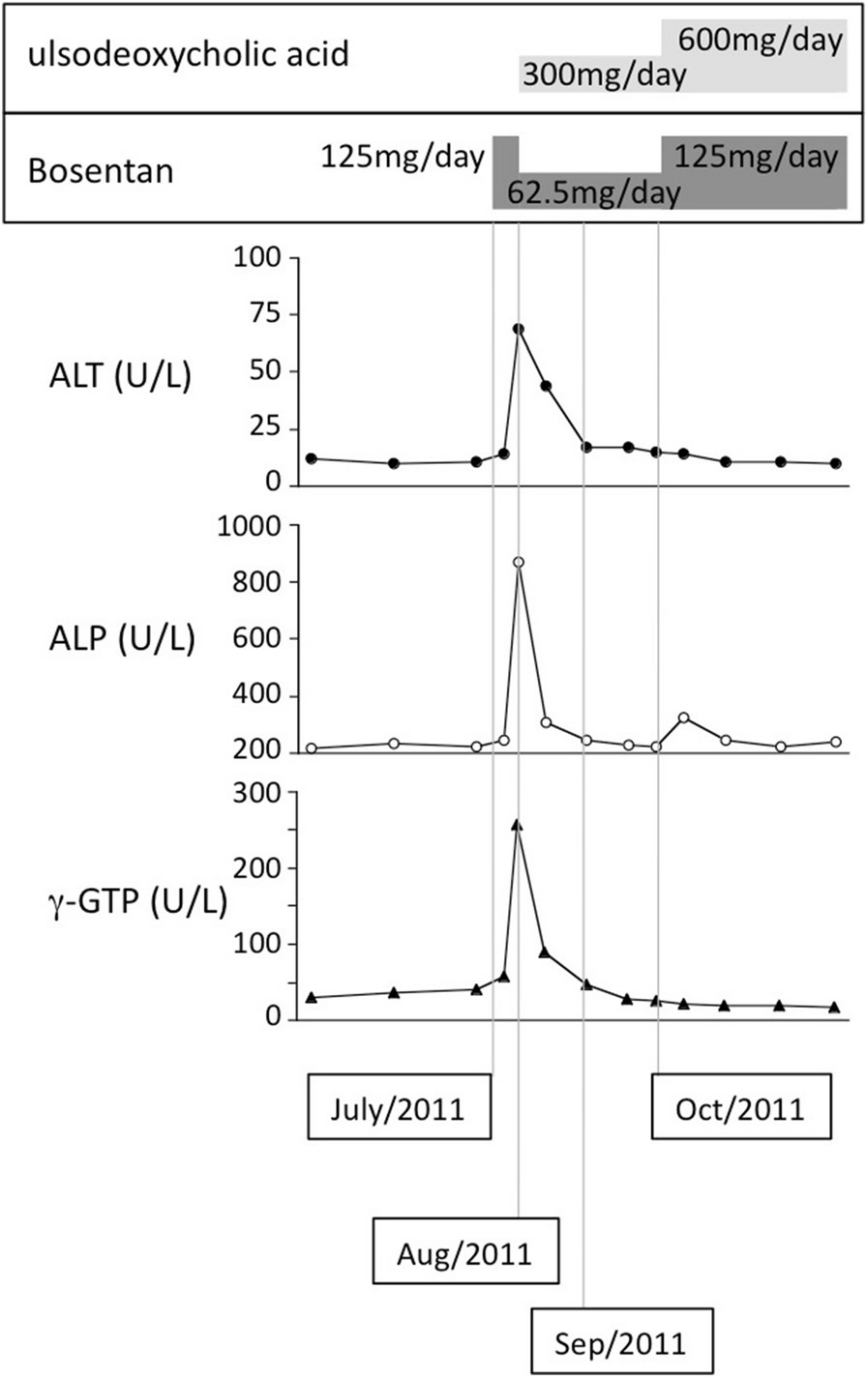
**Clinical course of Case 2.** Bosentan 125mg/day was started in July 2011; however, liver function abnormalities appeared after 3 weeks (August 2011). Bosentan was reduced to 62.5mg/day and ursodeoxycholic acid (300mg/day) was added simultaneously. Liver function abnormalities were normalized by September 2011. Bosentan was increased to the initial dose (125mg/day) concomitantly with increased ursodeoxycholic acid (600mg/day), and no liver function abnormalities appeared. Abbreviations: ALP, alkaline phosphatase; ALT, alanine aminotransferase; GGT, gamma-glutamyltransferase.

A 69-year-old Japanese woman who presented severe Raynaud’s phenomenon, hardening of the skin on her fingers and joint pain was diagnosed with systemic sclerosis. She was anti-centromere antibody and anti-mitochondria antibody positive. Echocardiography results showed 30mmHg of estimated right ventricular pressure in 2008. Betamethasone 0.25mg/day was introduced to control her joint pain. Raynaud’s phenomenon and dyspnea at exertion started to deteriorate from April 2011. Imaging findings did not show any abnormality in her lung field but echocardiography indicated an elevation of her estimated right ventricular pressure to 45mmHg, and we thus clinically diagnosed pulmonary arterial hypertension associated with connective tissue disease (WHO functional class III).Bosentan 125mg/day was started from July 2011, and this resulted in the improvement of breathlessness at exertion; however, after 3 weeks the results of her liver function tests revealed abnormal levels: elevation of ALT (1.9 times the normal upper limit), ALP (2.5 times the normal upper limit), and GGT (3.9 times the normal upper limit; Figure 2). No additional drugs were administered during the 2 months before the bosentan administration, and an ultrasound examination of her liver showed no morphologic abnormality at this time point. She was negative for both HBs Ag and HCV Ab. Bosentan was reduced to 62.5mg/day and ursodeoxycholic acid was introduced simultaneously at 300mg/day. Her liver function abnormalities normalized soon after the initiation of the combination therapy. Ursodeoxycholic acid was increased to 600mg/day and bosentan was increased to the initial dose of 125mg/day from October 2011. No abnormalities in her liver function were seen during 24 months of follow-up. Bosentan improved her estimated right ventricular pressure to 30mmHg and her subjective symptoms from WHO functional class III to II after the introduction of bosentan.

## Discussion

The results of the two patients’ liver function tests after initial bosentan monotherapy were abnormal, but not when ursodeoxycholic acid was combined as an adjunctive therapy with bosentan. This finding indicates that ursodeoxycholic acid is effective at preventing liver toxicity caused by bosentan, at least in the patients we treated.

Bosentan is mainly metabolized by cytochrome P450 enzymes CYP3A4 and CYP2C9 of the liver [10] but its pharmacokinetic variability is attributed to liver uptake transporters as well as metabolic enzymes, and the cause of liver injury is suggested to be an inhibition of the bile salt export pump [11]. Since ursodeoxycholic acid has been reported to induce an increased expression of CYP3A [12], the useful effect of ursodeoxycholic acid might be attributable to progression of the CYP3A-mediated metabolism of bosentan.

Even though a gradual increase in the dosage of bosentan is recommended, liver toxicity often occurs after its introduction or an increase in the dosage. If severe transaminitis (an over 8-fold increase in ALT/aspartate aminotransferase, AST, of the normal upper limit) occurs, bosentan treatment should be stopped immediately. However, if there is only mild transaminitis (less than an 8-fold increase in ALT/AST of the normal upper limit), bosentan can be reintroduced after dose reduction or interruption. Abnormal results of liver function tests are, in most cases, normalized after discontinuing bosentan, but we are often reluctant to restart bosentan. Our report suggests that it is, however, worth trying the reintroduction along with ursodeoxycholic acid, at least on the transient mild transaminitis. Pulmonary arterial hypertension still remains an incurable disease with a severe prognosis, and it is therefore important to start treatment with specific and effective drugs. Because bosentan is useful for the treatment of pulmonary arterial hypertension, it is desirable to avoid a dose reduction or discontinuation due to liver intolerance. Although there are alternative drugs (such as another endothelin receptor antagonist, ambrisentan, and phosphodiesterase type 5 inhibitors, sildenafil and tadalafil) with low liver toxicity approved for pulmonary arterial hypertension [13], it remains preferable to have more options available to us. Ursodeoxycholic acid is commonly used because of its safety and relatively low incidence of side effects, and its concomitant administration might help control the side effects of bosentan.

## Conclusions

In summary, the present report suggests that ursodeoxycholic acid has the potential to repress liver toxicity caused by bosentan. Thus, the combined use of oral ursodeoxycholic acid with bosentan is expected to be useful when liver toxicity emerges as a side effect of bosentan monotherapy.

## Consent

Written informed consent was obtained from the patients for publication of this case report and any accompanying images. Copies of the written consents are available for review by the Editor-in-Chief of this journal.

## Abbreviations

ALP: Alkaline phosphatase; ALT: Alanine aminotransferase; AST: Aspartate aminotransferase; GGT: Gamma-glutamyltransferase; HBs Ag: Hepatitis B surface antigen; HCV Ab: Hepatitis C virus antibody; WHO: World Health Organization.

## Competing interests

The authors declare that they have no competing interests.

## Authors’ contributions

TI supervised and wrote the manuscript. YO, YS, TN, HA, AT, TT, and SN participated in the patients’ therapy and helped to draft the manuscript. All authors read and approved the final manuscript.
